# Chronic cold exposure reprograms feeding-regulated LPL activity in white adipose tissues through hepatic ANGPTL3 and ANGPTL8

**DOI:** 10.1093/lifemeta/loae037

**Published:** 2024-10-16

**Authors:** Yiliang Zhang, Shengyang Zhou, Runming Zhao, Yingzhen Huang, Yan Wang

**Affiliations:** Hubei Key Laboratory of Cell Homeostasis, Department of Biochemistry, College of Life Sciences, TaiKang Center for Life and Medical Sciences, Wuhan University, Wuhan, Hubei 430072, China; Hubei Key Laboratory of Cell Homeostasis, Department of Biochemistry, College of Life Sciences, TaiKang Center for Life and Medical Sciences, Wuhan University, Wuhan, Hubei 430072, China; Hubei Key Laboratory of Cell Homeostasis, Department of Biochemistry, College of Life Sciences, TaiKang Center for Life and Medical Sciences, Wuhan University, Wuhan, Hubei 430072, China; Hubei Key Laboratory of Cell Homeostasis, Department of Biochemistry, College of Life Sciences, TaiKang Center for Life and Medical Sciences, Wuhan University, Wuhan, Hubei 430072, China; Hubei Key Laboratory of Cell Homeostasis, Department of Biochemistry, College of Life Sciences, TaiKang Center for Life and Medical Sciences, Wuhan University, Wuhan, Hubei 430072, China

## Abstract

**Graphical Abstract:**
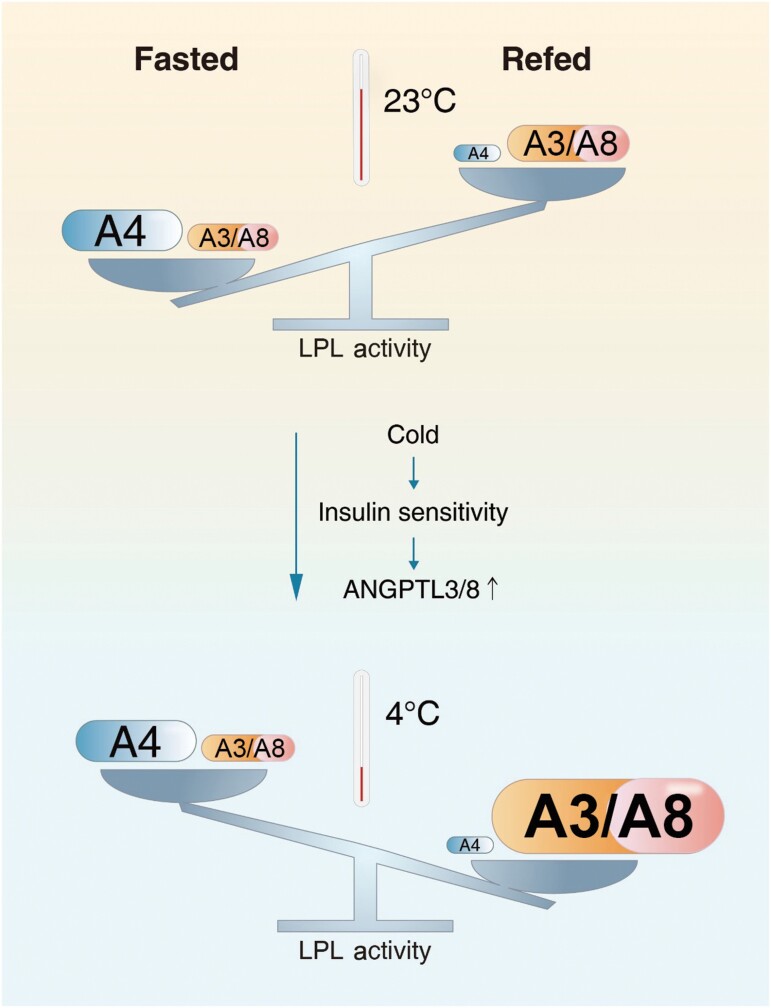

Lipoprotein lipase (LPL) mediates peripheral tissue triglyceride (TG) uptake. Hepatic ANGPTL3 (A3) and ANGPTL8 (A8) form a complex and inhibit LPL activity in the white adipose tissue (WAT) via systematic circulation. ANGPTL4 (A4) is expressed in WAT and inhibits LPL activity locally. Feeding increases hepatic A8 expression and increases its inhibition for WAT LPL activity together with A3, while feeding suppresses WAT A4 expression and releases its inhibition on LPL. At room temperature, the feeding-suppressed A4 overrides the feeding-increased A3/A8, resulting in increased LPL activity in WAT by food intake. Browning improves hepatic insulin sensitivity and increases postprandial A8 expression. The feeding-increased A3/A8 overrides the feeding-suppressed A4, resulting in suppressed LPL activity in WAT by food intake. This reprogrammed LPL regulation plays an important role in reprogramming TG metabolism during adipose tissue browning.

**Dear Editor,**

Chronic imbalance between energy intake and expenditure causes obesity and related metabolic complications [1]. The discovery of inducible brown adipocytes, known as beige adipocytes, has attracted broad interest as a potential way to increase energy expenditure and protect against obesity [2–5]. Beige adipocytes are interspersed within multiple depots of white adipose tissue (WAT), such as the subcutaneous WAT (scWAT) in mice [6]. During browning, the WAT is reprogrammed to brown adipose tissue (BAT)-like organs, both morphologically and functionally [7].

Circulation triglycerides (TGs) are an important metabolic fuel that supplies peripheral tissues through lipoprotein lipase (LPL)-mediated TG hydrolysis and tissue uptake [8]. Feeding increases LPL activity in WAT and suppresses its activity in energy-consuming tissues, such as the BAT, heart, and skeletal muscles, promoting the circulation of TG to the WAT for storage. Fasting reverses this regulation, promoting the circulation of TG to the energy-consuming tissues for energy production [8]. The reciprocal regulation of LPL activity in energy-storage tissues versus energy-consuming tissues plays a pivotal role in maintaining whole-body metabolic homeostasis. During browning, the WAT is reprogrammed from a fat-storage tissue to a fat-burning tissue. However, whether and how browning reprograms tissue LPL regulation in response to nutritional cues remains unknown.

The angiopoietin-like protein 3 (ANGPTL3, A3), 4 (ANGPTL4, A4), and 8 (ANGPTL8, A8) are three endogenous LPL inhibitors. A3 is strictly expressed in the liver, while A8 and A4 are primarily expressed in the liver and adipose tissues [9]. Feeding increases the expression of A8, which acts together with A3 to inhibit peripheral tissue LPL activity via systemic circulation [10–12]. Conversely, fasting increases the expression of A4 and inhibits LPL activity in adipose tissue locally [13, 14]. We recently found that the LPL activity in energy-consuming tissue is mainly regulated by circulating A3/A8, while WAT LPL activity is regulated by both circulating A3/A8 and locally expressed A4 [15]. A previous study reported that short-time cold exposure (< 10 days) suppresses A4 expression in BAT and increases the LPL activity for TG uptake [16]. However, whether A3, A4, and A8 are required for LPL regulation during WAT browning remains unknown.

Chronic cold exposure is a well-studied external stimulus for WAT browning. Consistently, a 12-week cold exposure dramatically increased the expression levels of uncoupling protein 1 (UCP1) and cell death-inducing DNA fragmentation factor alpha-like effector A (CIDEA) in scWAT (Supplementary Fig. S1a and b). Hematoxylin and eosin (H&E) staining also showed characteristics of browning [6] (Fig. 1a). Feeding increased LPL activity in the scWAT and decreased its activity in the heart and BAT, two representative energy-consuming tissues, in the mice housed at room temperature (RT). Whereas, a 12-week cold exposure reversed the feeding-regulated LPL activity in the scWAT, but not in the heart or BAT (Fig. 1b), suggesting that chronic cold exposure specifically reprograms the feeding-regulated LPL activity in the scWAT. Notably, chronic cold exposure dramatically increased LPL activity in the scWAT and BAT compared to that in mice housed at RT, consistent with their higher substrate demand for thermogenesis (Fig. 1b).

**Figure 1:**
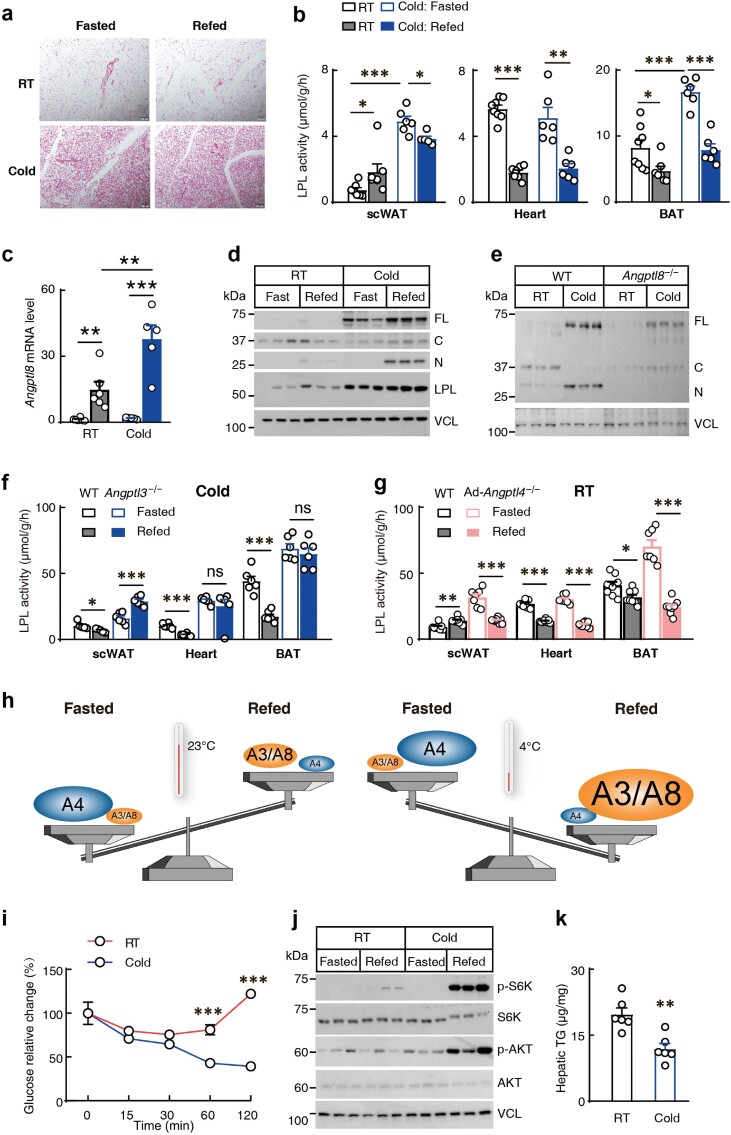
Chronic cold exposure reverses feeding-regulated LPL activity in scWAT through the hepatic insulin-ANGPTL8-ANGPTL3 axis. (a) Representative H&E staining of scWAT in mice. Scale bar, 100 μm. Mice were housed at RT or at 4°C (cold) for three months and samples were collected at fasting (fasted) or postprandial state (refed) (*n* = 6 mice/group, male, 20 weeks old). (b) Tissue LPL activity of mice housed at RT or at 4°C (cold) for 12 weeks (*n* = 6−8 mice/group, male, 20 weeks old). BAT, brown adipose tissue. (c) Hepatic *Angptl8* mRNA levels in mice housed at RT or at 4°C (cold) for three months (*n* = 5−6 mice/group, male, 20 weeks old). Samples were collected at fasting (fasted) or postprandial state (refed). (d) Representative immunoblots of indicated proteins in the scWAT of mice used in (a). FL, C, and N represent full-length, C-terminal, and N-terminal of ANGPTL3, respectively. VCL, Vinculin. (e) Representative immunoblots of ANGPTL3 in the scWAT of *Angptl8*^−/−^ mice and littermate control WT mice. Mice were housed at RT or at 4°C (cold) for three months and samples were collected at postprandial state (*n* = 3 mice/group, female, 20 weeks old). (f) Tissue LPL activity in *Angptl3*^−/−^ mice and littermate control WT mice. All mice were housed at 4°C for three months and samples were collected at fasting (fasted) or postprandial state (refed) (*n* = 6 mice/group, female, 19−27 weeks old), ns, not significant. (g) Tissue LPL activity in WT mice and Ad-*Angptl4*^−/−^ mice. All mice were housed at RT and samples were collected at fasting (fasted) or postprandial state (refed) (*n* = 7−8 mice/group, female, 9−21 weeks old). (h) Working model for the feeding-regulated LPL activity in scWAT before and after chronic cold exposure. The description is shown in the main text. (i) Insulin tolerance test in mice housed at RT or at 4°C (cold) for three months (*n* = 6 mice/group, male, 20 weeks old). Glucose levels are displayed as the percentage changes from time zero. (j) Representative immunoblots of insulin signaling pathway in mouse liver. Mice were housed at RT or at 4°C (cold) for three months and samples were collected at fasting (fasted) or postprandial state (refed). (*n* = 3 mice/group, male, 20−22 weeks old). AKT, protein kinase B; S6K, ribosomal protein S6 kinase. (k) Hepatic TG levels in mice housed at RT or at 4°C (cold) for three months. Samples were from the mice of the fasted group that was used in (a). Data are expressed as means ± SEM. ^*^P < 0.05, ^**^P < 0.01, ^***^P < 0.001.

A five-week cold exposure also dramatically increased the transcriptional levels of *Ucp1* and *Cidea* in the scWAT (Supplementary Fig. S1c), but to a lesser extent compared with mice subjected to a 12-week cold challenge (Supplementary Fig. S1a). The feeding-increased LPL activity was lost, but not reversed, in the scWAT of five-week cold-exposed mice (Supplementary Fig. S1d), suggesting that the feeding-regulated scWAT LPL activity is reprogrammed progressively during cold exposure. We subsequently used the 12-week cold exposure for further mechanistic studies.

It has been shown that scWAT LPL activity is regulated by both circulating A3/A8 and locally expressed A4 [15]. Chronic cold exposure dramatically increased postprandial *A8* mRNA levels in the liver and scWAT, with no significant changes in *A3* or *A4*  mRNA levels (Fig. 1c; Supplementary Fig. S1e and f). Chronic cold exposure also increased the transcriptional levels of *Lpl* in scWAT during both fasting and postprandial states (Supplementary Fig. S1f), consistent with the increased tissue LPL activity (Fig. 1b). Interestingly, feeding slightly increased *Lpl* mRNA levels in the scWAT of mice after chronic cold exposure (Supplementary Fig. S1f). However, feeding suppressed the LPL activity under this condition (Fig. 1b), suggesting that feeding regulates scWAT LPL activity mainly through post-transcriptional regulation.

Chronic cold exposure has no effect on the circulating levels of A3 (Supplementary Fig. S1g). However, it dramatically increased A3 levels in the scWAT, particularly for its full-length (FL) and N-terminal (N) forms during the postprandial state (Fig. 1d). We recently found that hepatic A8 promotes the binding of circulating A3 to peripheral tissue for LPL inhibition [15]. Consistently, the cold exposure-increased postprandial A3 binding in the scWAT was largely reduced in *Angptl8* knockout (*Angptl8*^−/−^) mice (Fig. 1e; Supplementary Fig. S1h), suggesting that chronic cold exposure increases A3 binding in the scWAT through increased hepatic A8 expression. Chronic cold exposure also increased the FL A3 binding in the scWAT during the fasting state (Fig. 1d). However, A3 is a poor LPL inhibitor during fasting when A8 expression level is low [11]. The function of the increased FL A3 in the fasting state remains unknown.

The increased A3 binding induced by chronic cold exposure suggests that A3/A8 may contribute more than A4 for LPL inhibition in the scWAT of cold-exposed mice. Thus, the feeding-increased A3/A8 may override the feeding-decreased A4, resulting in suppressed LPL activity in the scWAT following food intake (Fig. 1b). Consistently, for mice housed in chronic cold exposure, the feeding-suppressed scWAT LPL activity is reversed in *Angptl3* knockout (*Angptl3*^−/−^) mice (Fig. 1f), and in wild-type mice treated with an A3 inhibitor antibody (Supplementary Fig. S2a). For mice housed at RT, the feeding-increased scWAT LPL activity is reversed in adipocyte tissue-specific *Angptl4* knockout (Ad-*Angptl4*^−/−^) mice (Fig. 1g). These data indicate that the direction of feeding-regulated LPL activity in scWAT is determined by locally expressed A4 in mice housed at RT, and by circulating A3/A8 in mice exposed to chronic cold. At RT, the feeding-suppressed A4 expression overrides the feeding-increased A3/A8, resulting in increased LPL activity in the scWAT. Chronic cold exposure increases A3/A8 in scWAT without significant change in A4. The feeding-increased A3/A8 overrides the feeding-suppressed A4, resulting in suppressed LPL activity in the scWAT (Fig. 1h).

Notably, the feeding-regulated LPL activity was completely lost in the heart and BAT of *Angptl3*^−/−^ mice and in mice treated with an A3 inhibitor antibody (Fig. 1f; Supplementary Fig. S2a), suggesting that feeding regulates LPL activity in the heart and BAT primarily through A3, even in mice subjected to long-term chronic cold exposure. Previous studies also reported that short-term cold exposure (< 10 days) either increased [16] or decreased [17] *Angptl4* expression in the scWAT. However, we found that *Angptl4* mRNA levels did not change with long-term chronic cold exposure in either the fasting or postprandial state (Supplementary Fig. S1f). The function of ANGPTL4 in scWAT LPL regulation in response to cold exposure remains to be determined.

We recently found that feeding increases *Angptl8* transcription through the insulin-phosphoinositide 3-kinase (PI3K)-mechanistic target of rapamycin (mTOR) signaling pathway [15]. Chronic cold exposure did not significantly change insulin levels during either fasting or postprandial state (Supplementary Fig. S2b). However, chronic cold exposure dramatically increased whole-body insulin sensitivity (Fig. 1i). Consistently, chronic cold exposure enhanced the activity of the insulin signaling pathway (Fig. 1j; Supplementary Fig. S2c), and increased the transcriptional levels of fatty acid synthase (*Fasn*) and sterol regulatory element-binding protein-1c (*Srebp-1c*), two canonical insulin target genes in the liver and scWAT (Supplementary Fig. S2d and e). Body weight and hepatic TG levels were significantly decreased, which may be related to the improved insulin sensitivity in cold-exposed mice (Fig. 1k; Supplementary Fig. S2f).

Chronic cold exposure reprograms the feeding-regulated LPL activity in the scWAT along with its browning (Fig. 1). However, it is unclear whether the LPL regulation is reprogrammed through browning. Beige progenitor cells are more abundantly expressed in the inguinal WAT than in the gonadal WAT, making the former fat depot prone to browning [6]. We found that the feeding-increased LPL activity was abolished in the scWAT but was fully preserved in the epididymal WAT (epiWAT) of mice subjected to a five-week cold exposure (Supplementary Figs. S1d and S3a). However, longer-term cold exposure (12 weeks) also reversed the feeding-increased LPL activity in the epiWAT (Supplementary Fig. S3b), along with characteristics of adipose tissue browning (Supplementary Fig. S3c−e). As in the scWAT, a 12-week cold exposure dramatically increased A3 binding to the epiWAT in the postprandial state (Supplementary Fig. S3f). The feeding-suppressed LPL activity was reversed in the epiWAT of *Angptl3*^−/−^ mice after 12 weeks of cold exposure (Supplementary Fig. S3g), and the feeding-increased LPL activity was reversed in the epiWAT of Ad-*Angptl4*^−/−^ mice at RT (Supplementary Fig. S3h). These data suggest that long-term chronic cold exposure reprograms the feeding-regulated LPL activity in the epiWAT through the same mechanisms as that in the scWAT (Fig. 1h). The finding that the scWAT responds more quickly to cold exposure than the epiWAT in terms of feeding-regulated LPL activity, along with the abundance of beige adipocytes in these fat depots [6], suggests a role for browning in reprogramming the LPL activity in the feeding-regulated tissues. However, we cannot exclude other browning-independent effects during chronic cold exposure.

Efficient energy storage and utilization are key survival advantages for all living organs. In mammals, energy storage and utilization mainly take place in different organs. For example, more than 80% of energy is stored as TGs in mammals [18]. These TGs are stored primarily in the WAT and are utilized in energy-consuming tissues, such as the heart, skeletal muscles, and BAT. Circulating TGs are important fatty acid carriers for tissue oxidation and/or storage, and their uptake is tightly regulated by LPL located on the capillary endothelium of all peripheral tissues. Feeding increases LPL activity in the WAT and suppresses its activity in the energy-consuming tissues, diverting circulating TGs to adipose tissue for storage. Fasting reverses this regulation, diverting circulating TGs to energy-consuming tissues for utilization.

Three types of adipocytes have been identified in mammals: brown adipocytes, beige adipocytes, and white adipocytes. Brown adipocytes have high amounts of mitochondria with limited TG storage, while white adipocytes have very few mitochondria with large amounts of TG storage. Beige adipocytes have been found in different fat depots and can be induced to brown adipocyte-like cells in response to stimuli such as chronic cold exposure, a process known as browning [6]. Food intake regulates LPL activity in WAT and BAT reciprocally (Fig. 1b). However, whether and how feeding-regulated LPL activity is reprogrammed to meet their energy demands during WAT browning remains unknown. In the current study, we found that the feeding-increased LPL activity in WAT is reprogrammed gradually during chronic cold exposure, mainly through improved hepatic insulin sensitivity and A3/A8 activity.

Insulin serves as the cornerstone for anabolism and energy storage. It is well known for promoting lipid synthesis and suppressing lipolysis in adipose tissue [19, 20]. Recently, we uncovered the key role of insulin in promoting circulating TGs to replenish WAT for storage by suppressing LPL activity in energy-consuming tissues through hepatic A3/A8 [11, 12]. Inducible thermogenesis is crucial for defending against chronic cold exposure in mammals [7]. Lipids are the preferred substrates for thermogenesis [21]. Consistently, LPL activity in scWAT, epiWAT, and BAT is dramatically increased during chronic cold exposure, especially at the fasting state (Fig. 1b; Supplementary Fig. S3a and b). However, besides cold exposure, mice must cope with fluctuations in food availability simultaneously. Efficient energy storage is even more crucial in this harsh environment when food is not plentiful. The reprogrammed feeding-regulated LPL activity during WAT browning provides a way to enhance proper TG storage during chronic cold exposure. Our current study revealed another important role of insulin in maintaining whole-body homeostasis.

Muscle-specific LPL expression in *Lpl* knockout mice leads to decreased TG uptake into adipose tissue, accompanied by increased *de novo* lipogenesis, increased fatty acid oxidation, and characteristics of browning [22]. Both *Angptl3*^−/−^ and *Angptl8*^−/−^ mice show decreased TG-derived fatty acid uptake in the WAT, including the scWAT [11, 12]. Both mouse models exhibit increased rectal temperature, with A3 and A8 double knockout mice having even higher body temperature and enhanced scWAT browning [23]. These data suggest that LPL-mediated fatty acid uptake may also function as a signal to constrain WAT browning. A3 and A8 inhibit LPL activity and TG-derived fatty acid uptake in the scWAT during the postprandial state, indicating that A3/A8 may contribute to the browning of scWAT during chronic cold exposure. To fully elucidate the functions of A3/A8 in WAT browning, new animal models will be needed to specifically disrupt A3/A8 function in WAT, as hepatic A3/A8 functions via systemic circulation, and the whole-body knockout mice have dramatically increased LPL activity in energy-consuming tissues, which compete with the WAT for circulating TGs.

## Supplementary Material

loae037_suppl_Supplementary_Figures

## Data Availability

All data generated or analyzed during this study are included in this article and its supplementary information files.
