# Supplementary material for: Chronic cold exposure reprograms feeding-regulated LPL activity in white adipose tissues through hepatic ANGPTL3 and ANGPTL8
Source: Life Metab. 2024 Oct 16;4(1):loae037. doi: 10.1093/lifemeta/loae037 (PMC11770819; doi:10.1093/lifemeta/loae037)
Supplement: loae037_suppl_Supplementary_Figures [file loae037_suppl_supplementary_figures.docx]

Supplementary Information for

**Chronic cold exposure reprograms feeding-regulated LPL activity in white adipose tissues through hepatic ANGPTL3 and ANGPTL8**

Yiliang Zhang, Shengyang Zhou, Runming Zhao, Yingzhen Huang, Yan Wang*

Hubei Key Laboratory of Cell Homeostasis, Department of Biochemistry in College of Life Sciences, TaiKang Center for Life and Medical Sciences, Wuhan University, Wuhan, Hubei 430072, China.

*Corresponding author. Hubei Key Laboratory of Cell Homeostasis, College of Life Sciences, TaiKang Center for Life and Medical Sciences, Wuhan University, Wuhan, Hubei 430072, China. E-mail: [Wang.y@whu.edu.cn](mailto:Wang.y@whu.edu.cn)

**Supplemental Materials**

**Materials**

We purchased insulin from Novo Nordisk (11061-68-0), 9,10-[^3^H]triolein from PerkinElmer (NET431005MC), fatty acid-free bovine serum albumin (BSA) from Sangon Biotech (A602448), heparin from Beijing Wokai Biotechnology (A47476), and insulin ELISA kit from Ezassay (MS100). ANGPTL3 inhibitory antibody was described previously [[1](#_ENREF_1)].

**Animals:**

C57BL/6J wild-type (WT) mice were obtained from the Central Disease Control of Hubei province, China. The adipocyte-specific *Angptl4* knockout (Ad-*Angptl4*^−/−^) mice and *Angptl8* knockout (*Angptl8*^−/−^) mice were described previously [[1](#_ENREF_1)]. Littermate control mice were used for all studies unless otherwise indicated. Mice were housed in the specific pathogen-free barrier facility with controlled environment (12-h light/12-h dark daily cycle, 23 ± 1 °C, 60−70% humidity) at Wuhan University. All animal protocols were approved by the Institutional Animal Use and Care Committee of College of Life Sciences, Wuhan University. All animals were fed with a standard chow diet with free access to food and water unless otherwise indicated.

For chronic cold exposure, mice were divided into two groups randomly. For cold exposure group, mice were first put at 18 °C for one week and then switched to 4 °C for different time. Control mice were housed at room temperature (RT) side by side. Each mouse was housed individually with free access to food and water. For fasting and refeeding experiments, mice were first synchronized with food intake for three days by daytime fasting (7:00 a.m. to 5:00 p.m.) and nighttime feeding (5:00 p.m. to 7:00 a.m.). On day 4, samples were collected at 8:00 p.m, which is 3 h after refeeding for the refed group and 13 h after fasting for the fasted group.

**Blood chemistry**

Blood was collected by eye bleeding after being anesthetized with isoflurane. Blood glucose was measured using glucometer (Contour; Bayer) during blood collection. Whole blood was allowed to stand at RT for 60 min. Serum was isolated by centrifugation at 3,500 *g* for 10 min at 4°C. Triglycerides (TGs) and cholesterol were measured using enzymatic kits from Shanghai Kehua Bio-Engineering Co. Non-esterified fatty acids were measured using HR Series NEFA-HR Kit (Wako).

**Insulin tolerance test**

Insulin tolerance test was performed following a 4-h fasting by interphalangeal injection of insulin (0.75 U/kg body weight). Blood glucose levels were measured by tail vein bleeding at 0, 15, 30, 60, and 120 min after injection.

**Hepatic lipid extract.**

Hepatic lipids were extracted as described previously [[2](#_ENREF_2)]. Briefly, 25−50 mg frozen liver was homogenized with 1 mL Folch solution. Then 200 μL PBS was added and the samples were vortexed briefly. The mixture standed at RT for 10 min and then centrifuged at 1500 *g* for 10 min. The lower phase was collected and dried by nitrogen gas completely. The pellets were suspended with ethanol and TG content was measured with an enzymatic kit. Hepatic TG levels were expressed as per mg of wet tissues.

**Tissue lipoprotein lipase (LPL) activity assay**

Tissue LPL activity was measured as described previously [[2](#_ENREF_2)]. Briefly, fresh tissues were collected and minced in cold DMEM medium (Life Technologies, Grand Island, USA. 12800-082) with fatty acids-free BSA (2%) and heparin (2 U/mL) from mice anesthetized with isoflurane. After slow rotation at 37°C for 30 min, the mixture was spin down at 6,000 *g* for 5 min. The supernatant was collected and snapped frozen at liquid nitrogen and saved at −80^o^C for further analysis. Triglyceride lipase activity was measured using a glycerol-stabilized emulsion composed of 9,10-[^3^H]triolein, triolein, and phosphatidylcholine. LPL activity is expressed as the rate of free fatty acid release.

**Immunoblotting analysis**

Sample preparation and immunoblotting analysis were performed in the same way as previously described [[1](#_ENREF_1)]. Mice were perfused with ice-cold PBS through the left ventricle after anesthetizing with isoflurane. Tissues were harvested and snap-frozen in liquid nitrogen and saved at −80^o^C for further analysis. Tissue lysate was prepared by homogenizing in lysis buffer (25 mmol/L Tris, pH 7.4, 25 mmol/L glycine, 150 mmol/L NaCl, 5 mmol/L EDTA, and 1% triton X-100) with EDTA-free protein inhibitor cocktail (Selleck, B14001) and EDTA-free Phosphatase Inhibitor Cocktail (AbMole Bioscience, M7528). The serum was diluted for 20 folds with saline before adding sample buffer. A 4 × non-reducing sample buffer (124 mM Tris-HCL, pH 6.8, 4% SDS (W/V), 50% glycerol (V/V), bromophenol blue) was used for all samples unless otherwise indicated.

The following primary antibodies were used in the current study: Anti-ANGPTL3 antibody (AF136, R&D Systems, Minneapolis, USA), Anti-VCL antibody (A2752, ABclonal), Anti-LPL antibody (AF7197, R&D Systems), Anti-AKT antibody (88800, Cell Signaling Technology), Anti p-AKT antibody (4060, Cell Signaling Technology), Anti-S6K antibody (14485, Proteintec), Anti p-S6K antibody (97596, Cell Signaling Technology), Anti-Ucp1 antibody (A21979, ABclonal), and Anti-CideA (100879-T32, Sino Biological).

**Real-time PCR analysis**

Total RNA was extracted with TRI Reagent (Sigma-Aldrich, T9424) and was quantified with NanoDrop (Thermo Scientific). Total RNA (2 μg) were subjected to reverse transcription with a commercial Kit (Yeasen, China, 11121ES60). The Real-time PCR was performed with SYBR Green master mix (Yeasen, China, 11201ES08) together with indicated primers for each gene. *36B4* was used as an internal control. The average expression levels of the control group were set to 1. All primer sequences will be provided upon request**.**

**Hematoxylin and eosin (H&E) staining**

Fresh tissues were fixed in paraformaldehyde (4%) overnight, followed by washing with PBS, and processed for paraffin embedding. Then the tissues were sectioned to a thickness of 5 μm and stained with H&E. Stained sections were subjected to light microscopy for image acquisition (Olympus DP80, ×10 magnification).

**Data analysis**

All data are expressed as mean ± SEM and *P* values were calculated using Student’s *t*-test in GraphPad Prism 8.0.1 unless otherwise indicated.

**Supplementary Figure S1**


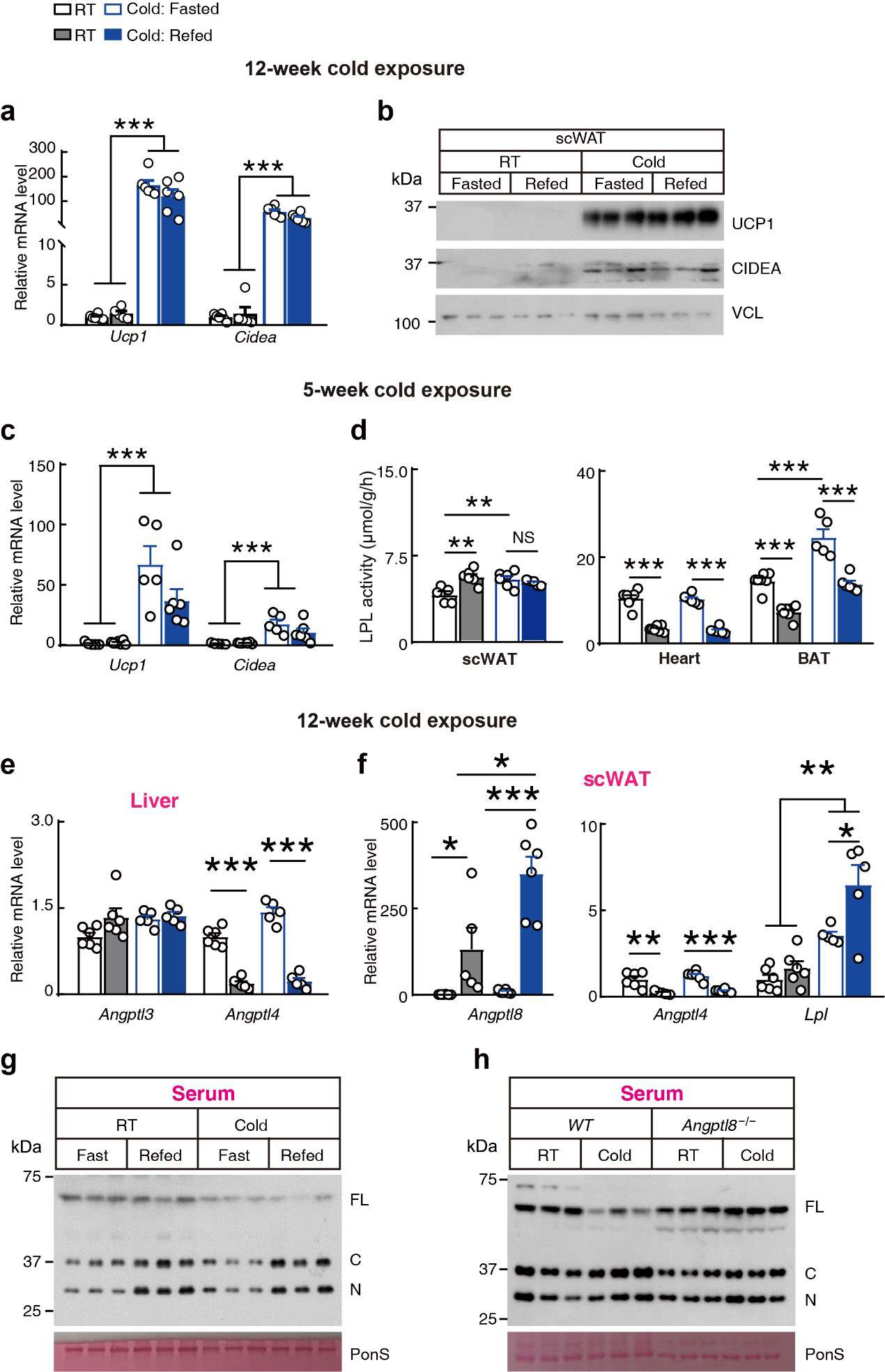


**Supplementary Figure S1** (a) Relative mRNA levels of *Ucp1* and *Cidea* in subcutaneous white adipose tissue (scWAT). Mice were housed at room temperature (RT) or at 4°C (Cold) for three months and samples were collected at fasting (Fasted) or postprandial (Refed) state (*n* = 6 mice/group, male, 20 weeks old). (b) Representative immunoblots of scWAT in mice used in a. (c) Gene expression levels in the scWAT of mice that were housed at RT or subjected to chronic cold exposure for five weeks (*n* = 6−7 mice/group, male, 13−15 weeks old). (d) Tissue LPL activity in mice used in c. (e and f) Relative mRNA levels in tissues of mice housed at RT or at 4°C (Cold) for three months, corresponding to mice used in Figure 1c. (g and h) Representative immunoblots of serum in mice used in Figure1d and 1e, respectively. FL, C, and N represent full-length, C-terminal, and N-terminal of ANGPTL3, respectively. PonS, ponceau S. Data are expressed as means ± SEM. ^*^*P* < 0.05, ^**^*P* < 0.01, ^***^*P* < 0.001.

**Supplementary Figure S2**


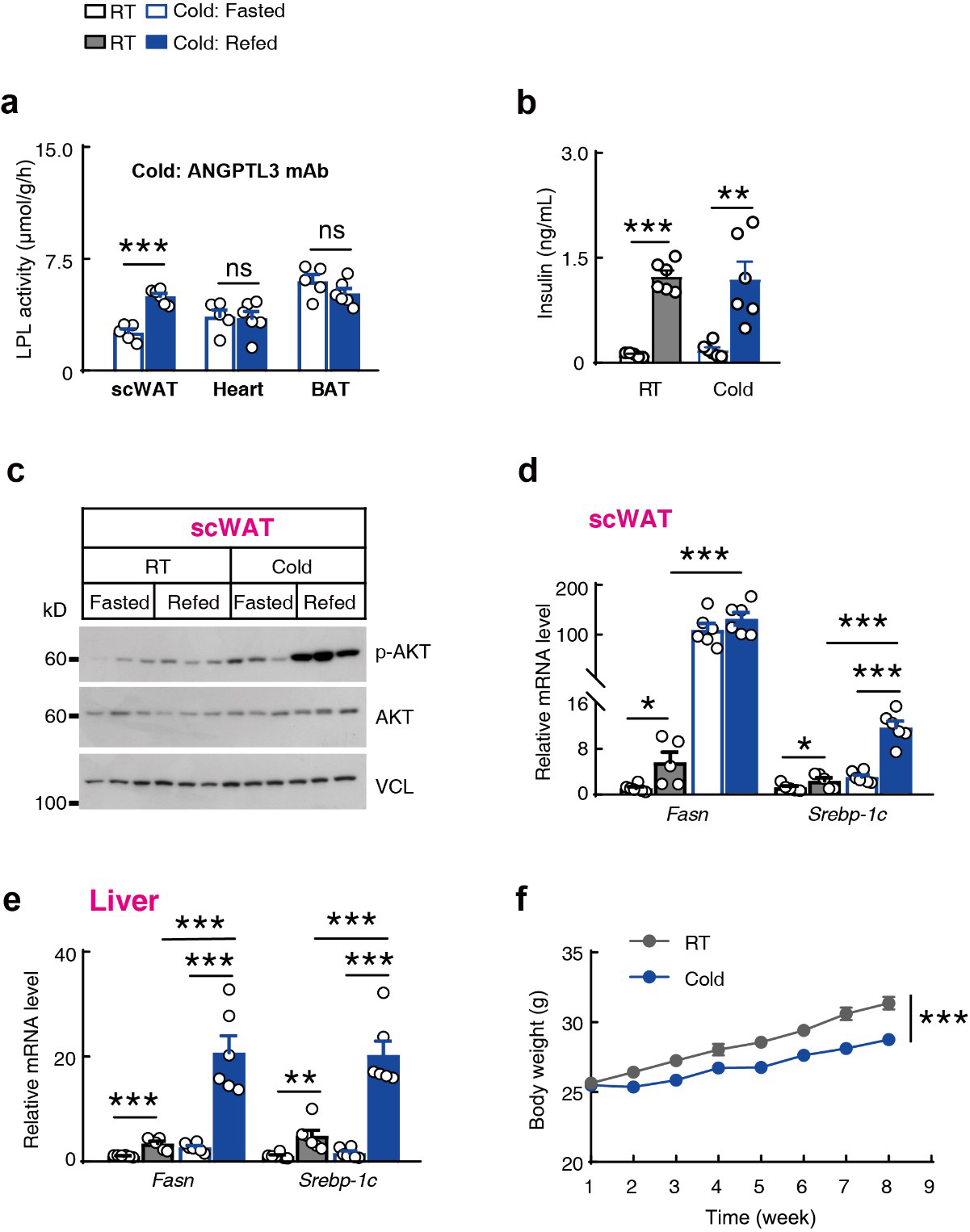


**Supplementary Figure S2** (a) Tissue LPL activity in cold-exposed mice treated with ANGPTL3 inhibitory antibody (ANGPTL3 mAb). Mice were housed at 4°C for 12 weeks. ANGPTL3 mAb was infused through tail vein for another four days and the tissues were collected at fasting (Fasted) or postprandial (Refed) state (*n* = 5−6 mice/group, male, 20 weeks old. ns, non-significant). (b) Serum insulin levels of mice used in Figure 1a. (c) Representative immunoblots of scWAT in mice used in Figure 1j. (d and e) Relative mRNA levels in scWAT and liver of mice used in Figure 1a. (f) Body weight of mice housed at RT or at 4°C (Cold) for indicated time. Cold exposure was started at the age of eight weeks (*n* = 24 mice/group, male). Data are expressed as means ± SEM. ^*^*P* < 0.05, ^**^*P* < 0.01, ^***^*P* < 0.001. All abbreviations are the same as in Figure 1.

**Supplementary Figure S3**


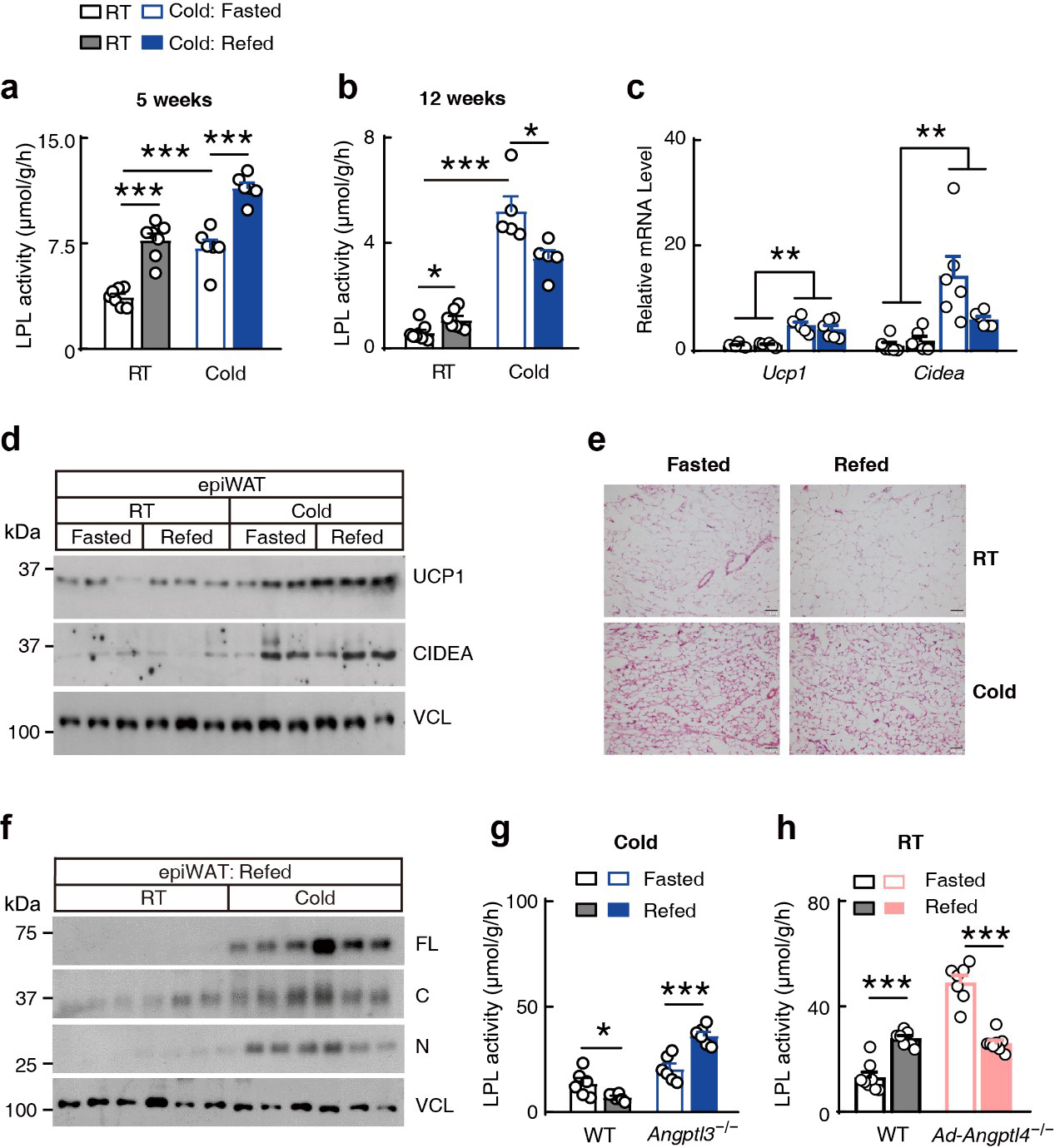


**Supplementary Figure S3** Chronic cold exposure reprograms the feeding-regulated LPL activity in epididymal white adipose tissues (epiWAT) like that in subcutaneous white adipose tissue. (a) LPL activity in epiWAT of mice housed at RT or at 4°C (Cold) for five weeks. The mice used were corresponding to that used in Supplementary Figure S1c. (b) LPL activity in epiWAT of mice housed at RT or at 4°C (Cold) for 12 weeks. The mice used were corresponding to that used in Figure 1b. (c and d) Expression levels of UCP1 and CIDEA in epiWAT of mice used in Figure 1a. (e) Representative H&E staining of epiWAT in mice used in d (Scale bar, 100 μm). (f) Representative ANGPTL3 immunoblots in epiWAT of mice at postprandial state (Refed). Mice were housed at RT or at 4°C (Cold) for 12 weeks (*n* = 6 mice/group, male, 20 weeks old). FL, C, and N represent full-length, C-terminal, and N-terminal of ANGPTL3, respectively. VCL, Vinculin. (g) LPL activity in epiWAT of *Angptl3* knockout (*Angptl3*^−/−^) mice and littermate control wild-type (WT) mice. All mice were housed at 4°C for 12 weeks. The mice were corresponding to that used in Figure 1f. (h) LPL activity in epiWAT of wild-type (WT) mice and adipose tissue-specific *Angptl4* knockout (Ad-*Angptl4*^-/-^) mice. All mice were housed at RT. The mice were corresponding to that used in Figure 1g. Data are expressed as means ± SEM. ^*^*P* < 0.05, ^**^*P* < 0.01, ^***^*P* < 0.001. All abbreviations are the same as in Figure 1.

**References**

1. Liu X, Zhang Y, Han B *et al.*, Postprandial exercise regulates tissue-specific triglyceride uptake through angiopoietin-like proteins*.* *JCI Insight*, 2024;**9**:e181553.

2. Wang Y, McNutt MC, Banfi S *et al.*, Hepatic ANGPTL3 regulates adipose tissue energy homeostasis*.* *Proc Natl Acad Sci U S A*, 2015;**112**: 11630−5.
